# Editorial: Bioinformatics in Microbiota

**DOI:** 10.3389/fmicb.2020.00100

**Published:** 2020-02-05

**Authors:** Xing Chen, Hongsheng Liu, Qi Zhao

**Affiliations:** ^1^School of Information and Control Engineering, China University of Mining and Technology, Xuzhou, China; ^2^School of Life Science, Liaoning University, Shenyang, China; ^3^College of Computer Science, Shenyang Aerospace University, Shenyang, China

**Keywords:** bioinformatics, human microbiota, disease, big data, computational method

Microbiota are a group of microscopic organisms with simple structures and include bacteria, fungi, viruses, and others. Increasing numbers of biological experiments have shown that microbiota play a significant role in the occurrence and development of human diseases. Understanding the relationship between the microbiota and the host disease can be very useful in the treatment of complex diseases, such as inflammatory bowel disease, diabetes, and so on. However, using traditional wet experimental methods to identify microbe-disease associations is costly and time-consuming. During recent years, benefitting from the rapid development of artificial intelligence, machine learning, and new complex network techniques have been developed to work for the big data generated from human microbiome experiments. This Research Topic explores the potential for these computational methods applied to the research of human microbiota.

We are pleased to note that our Research Topic has attracted contributions from many highly regarded researchers in this field around the world, including from China, the USA, Spain, Chile, Korea, and India. We received 75 submissions, 39 of which were accepted for publication after rigorous reviews. We have further categorized these manuscripts into four subtopics with the Research Topic.

There are nine papers discussing the relationship between microbe and disease in the first part of this special issue. Zhou S. et al. examined the correlations between the gene expression levels of defensins and the viral and bacterial loads in the blood on a longitudinal, precision medicine study of a severe pneumonia patient infected by influenza A H7N9 virus. They showed that DEFB116 and DEFB127 are positively correlated and DEFB108B and DEFB114 are negatively correlated to the bacterial load. He B.-S. et al. proposed a novel predictive model of graph regularized non-negative matrix factorization for human microbe-disease relationship prediction based on known microbe-disease associations, Gaussian interaction profile kernel similarity for microbes and diseases, and symptom-based disease similarity. Wang et al. proposed a novel low-rank matrix completion model named MCAAS to infer antigenic distances between antigens and antisera based on partially revealed antigenic distances, virus similarity based on HA protein sequences, and vaccine similarity based on vaccine strains. Peng et al. established a model of adaptive boosting for human microbe-disease association prediction (ABHMDA) to reveal the associations between diseases and microbes. Chen J. et al. made a patient level analysis between abscess and healthy periodontium, which showed that *P. gingivalis*, and *Prevotella* spp. including *P. intermedia* were found to be dominant in the abscess of some patients compared to those of healthy periodontium, based on 16S rDNA metagenomic sequencing. Niu et al. introduced an *in silico* model named RWHMDA to predict underlying microbe-disease associations. Both cross-validation and case studies on asthma, type 2 diabetes, and Crohn's disease revealed the reliability of RWHMDA. Li H. et al. proposed a novel prediction model called BWNMHMDA to accelerate the process of inferring potential microbe-disease associations, in which, the core idea is to construct a weighted bidirectional microbe-disease association network and then convert it into a matrix for correlation probability calculation. Qu J. et al. put forward the matrix decomposition and label propagation for human microbe-disease association prediction (MDLPHMDA) on the basis of the dataset of known microbe-disease associations collected from the database of HMDAD, the Gaussian interaction profile kernel similarity for diseases and microbes, and disease symptom similarity. Zhou W. et al. showed that the changes in the fecal microbiome were associated with age and disease progression in Zucker diabetic fatty rats.

Nine papers included in second part are focused on gut microbiota. Li W. et al. applied both Hubbell's and Sloan's neutral theory models to test the influence of obesity on the gut microbiome assembly from both community and species perspectives. Lai et al. investigated the effects of dietary perfluorooctane sulfonic acid (PFOS) exposure on gut microbiota in adult mice and examined the induced changes in animal metabolic functions. Zeng et al. characterized the microbial biogeographical characteristics in the GIT of a red panda using high-throughput sequencing technology. Ma W. et al. treated healthy mice with metformin and found that metformin could indeed prominently affect gut microbiome structure in healthy mice. Medina et al. compared the composition of the human gut microbiota of obese and lean people from six different regions and showed that the microbiota compositions in the context of obesity were specific to each studied geographic location. Yin and Xia firstly adopted a Silverman's test on the original results of the hybrid model, next using this strategy to reanalyze a dataset of HIV-related human gut microbiome in order to find HIV-specific changes in the assembly of gut microbial communities. Dai et al. constructed metabolic dependency networks using gut microbiota datasets of common enteric diseases including IBD and CRC, and revealed unappreciated interaction patterns of disease-enriched bacteria and probiotics. Ai et al. studied the microbial community structure of a CRC metagenomic dataset of 156 patients and healthy controls, and analyzed the diversity, differentially abundant bacteria, and co-occurrence networks. Quan et al. performed a comparative analysis of the fecal microbiota in DLY pigs with polarizing FE using 16S rRNA gene sequencing and shotgun metagenomic sequencing.

There are 12 papers with machine learning techniques applied in the research of microbiomes. Xiao et al. proposed a predictive framework to exploit sparse and clustered microbiome signals using a phylogeny-regularized sparse regression model. Xiong et al. developed a stacked ensemble model PredT4SE-Stack to predict T4SEs, which utilized an ensemble of base-classifiers implemented by various machine learning algorithms, to generate outputs for the meta-classifier in the classification system. Chaudhari et al. developed PanGFR-HM, a novel dynamic web-resource that integrates genomic and functional characteristics of 1,293 complete microbial genomes available from the Human Microbiome Project. He W. et al. collected the ncDNA benchmark dataset of *Saccharomyces cerevisiae* and developed a support vector machine-based predictor, called Sc-ncDNAPred, for predicting ncDNA sequences. Zhang et al. presented a computational method to identify m^6^A sites in the *E.coli* genome by encoding the RNA sequences using nucleotide chemical properties and accumulated nucleotide frequency. Manavalan et al. described a novel computational method for predicting PVPs, called PVP-SVM, and utilized the available PVPs sequences to develop the method. Hao et al. reviewed three representative genome-scale cellular networks: GMN, TRN, and STN, and discussed the integration of the three types of networks. Qu K. et al. discussed the current application of machine learning methods in the microbiome. They reported that machine learning is widely used in microbiological research, and that it has focused on classification problems and analysis of interaction problems. Khan et al. developed an approach for prediction of the global burden of tuberculosis based on artificial neural networks. Ru et al. proposed a random forest method to classify bacteriophage virion proteins and non-phage virion proteins. Wei and Zhang presented a novel dynamic multi-seeds clustering method (namely DMSC) to pick operational taxonomic units. Chung et al. developed a statistical test-based method to determine the reference spectrum when dealing with alignment of mass spectra datasets, and constructed machine learning-based classifiers for categorizing different strains of *S. haemolyticus*.

Other studies are categorized as the fourth part of our special issue. There are nine papers in total in this part. Ma Z. et al. reconstructed a genome-scale metabolic model (GSMM) of a *Ganoderma lucidum* strain, and applied this model to elucidate detailed physiological characteristics and production of extracellular polysaccharide in this species. Chen Y. X. et al. isolated 65 rhizobial strains from faba bean, then studied their plant growth promoting ability with nitrogen free hydroponics, genetic diversity with clustered analysis of combined ARDRA and IGS-RFLP. Ma and Li analyzed the scaling of semen microbiome diversity across individuals with diversity-area relationship analysis, a recent extension to classic species-area relationship law in biogeography and ecology. Tamames et al. proposed a fully automatic pipeline (SqueezeMeta) for metagenomics/metatranscriptomics, covering all steps of the analysis. Nagpal et al. presented iVikodak, a multi-modular web-platform that hosts a logically inter-connected repertoire of functional inference and analysis tools, coupled with a comprehensive visualization interface. Kioroglou et al. performed metataxonomic analysis of two types of mock community standards with the same microbial composition for evaluating the effectives of QIIME balanced default parameters on a variety of aspects related to different laboratory and bioinformatic workflows. Burcham et al. monitored bacterial community structural and functional changes taking place during decomposition of the intestines, bone marrow, lungs, and heart in a highly controlled murine model. Li and Ma investigated the microbiome diversity scaling over space by analyzing the diversity-area relationship, which is an extension to classic species-area relationship law in biogeography. Kuntal et al. presented Web-gLV—a GUI based interactive platform for generalized Lotka-Volterra (gLV) based modeling and simulation of microbial populations.

Finally, we want to thank all the authors who contributed their original work to our special issue and the reviewers for their valuable comments. We would like to express our sincere gratitude to the Specialty Chief Editor, Dr. Matthias Hess and Dr. George Tsiamis, and also the editorial office of Frontiers in Microbiology, for their excellent support and providing us with this opportunity to organize this hot topic issue successfully.

## Author Contributions

QZ and XC wrote and revised the manuscript. HL gave some helpful suggestions.

### Conflict of Interest

The authors declare that the research was conducted in the absence of any commercial or financial relationships that could be construed as a potential conflict of interest.

